# Dedifferentiated Schwann cells promote perineural invasion mediated by the PACAP paracrine signalling in cervical cancer

**DOI:** 10.1111/jcmm.17897

**Published:** 2023-10-13

**Authors:** Guoqiang Chen, Zhen Zheng, Hao Sun, Jiahao You, Jing Chu, Jinghai Gao, Lei Qiu, Xiaojun Liu

**Affiliations:** ^1^ Department of Obstetrics and Gynecology Second Affiliated Hospital of Naval Medical University Shanghai China; ^2^ Department of Gynecology, The People’s Hospital of Baoan Shenzhen The Second Affiliated Hospital of Shenzhen University Shenzhen China; ^3^ Department of Obstetrics and Gynecology, Shanghai Jiao Tong University Affiliated Sixth People’s Hospital Shanghai Jiao Tong University School of Medicine Shanghai China; ^4^ School of Pharmacy Naval Medical University Shanghai China

**Keywords:** cervical cancer, dedifferentiated, perineural invasion, pituitary adenylate cyclase‐activating polypeptide, Schwann cells

## Abstract

Perineural invasion (PNI) has emerged as a key pathological feature and be considered as a poor prognostic factor in cervical cancer. However, the underlying molecular mechanisms are largely unknown. Here, PNI status of 269 cervical squamous cell carcinoma and endocervical adenocarcinoma (CESC) samples were quantified by using whole‐slide diagnostic images obtained from The Cancer Genome Atlas. Integrated analyses revealed that PNI was an indicative marker of poorer disease‐free survival for CESC patients. Among the differentially expressed genes, ADCYAP1 were identified. Clinical specimens supported that high expression of PACAP (encoded by ADCYAP1) contributed to PNI in CESC. Mechanistically, PACAP, secreted from cervical cancer cells, reversed myelin differentiation of Schwann cells (SCs). Then, dedifferentiated SCs promoted PNI by producing chemokine FGF17 and by degrading extracellular matrix through secretion of Cathepsin S and MMP‐12. In conclusion, this study identified PACAP was associated with PNI in cervical cancer and suggested that tumour‐derived PACAP reversed myelin differentiation of SCs to aid PNI.

## 1 INTRODUCTION

Mounting evidence has indicated that the nervous system plays an essential role in cancer progression. Tumour cells release various neurotrophic factors to promote their own innervation.^1^, ^2^, ^3^ In turn, infiltrating nerves also mediate various malignant biological processes of the tumour.^4^ Such discoveries make identification of predictive markers and mechanisms of cancer neuroscience an area of emerging research. Most reports about the interactions between solid tumours and the nervous system have described perineural invasion (PNI). PNI is defined as the presence of cancer cells that cover the nerve by at least 33% of its circumference or reside within any of its three layers of nerve sheath.^5^ PNI is a poorly understood process by which tumour cells invade nerves and metastasize. It is a major pathological feature of many malignant tumours including prostate cancer,^6^ pancreatic cancer,^7^ and cervical cancer.^8^ For these malignancies, PNI is a marker of a poor prognosis and a predictor of poor survival.^5^ PNI is a major cause of recurrence and metastasis of cervical cancer after surgical resection. It can occur in the absence of lymphatic or vascular invasion,^5^ and PNI often predicts a poor prognosis of patients with early cervical cancer.^9^ Pelvic malignancies most frequently invade L5–S1 spinal nerves and the sciatic nerve.^10^ These nerves provide a favourable sanctuary for tumour cells to avoid immune clearance and death induced by chemotherapeutic drugs. Tumour cells can use pelvic autonomic nerves as conduits and spread from the end cervix to pelvic bone and pelvic muscle tissue. Clinical studies have found that the detection rate of PNI in cervical cancer is 7.0%–35.1%,^11^ and 5‐year overall survival was significantly lower in PNI(+) cervical cancer patients than in PNI(−) cervical cancer patients (51.1% [95% CI 38.0–64.2] vs. 75.6% [95% CI 67.8–83.4]; *p* = 0.001).^8^ Additionally, compared with their counterparts, PNI(+) cervical cancer patients were also more likely to have positive lymph nodes, cervical tunica adventitia invasion and deep outer cervix stromal invasion.^12^

It was previously believed that PNI is due to the potential gap between nerve fascicles, so‐called low resistance channels, which are conducive to the invasion and spread of tumour cells.^13^ However, recent studies have shown that PNI is the result of interactions between nerve, tumour and stromal cells. Tumour cells promote own innervation by recruiting local nerves or neural progenitor cells,^3^, ^14^, ^15^ and then crosstalk between neurons and tumour cells also leads to PNI.^16^, ^17^, ^18^ However, the mechanism of PNI in cervical cancer remains unclear.

In this study, we used large‐scale cervical squamous cell carcinoma and endocervical adenocarcinoma (CESC) diagnostic images and molecular profiles from The Cancer Genome Atlas (TCGA) database, found that the level of pituitary adenylate cyclase‐activating polypeptide (PACAP) in tumour tissues was positively related to the PNI status in cervical cancer. PACAP, encoded by the *ADCYAP1* gene, is an endocrine neuropeptide and member of the vasoactive intestinal peptide (VIP)/secretin/glucagon family.^19^ PACAP binds with high affinity to three receptors (PAC1R, VIPR1 and VIPR2). PAC1R specifically binds PACAP, and VIPR has equal affinity for PACAP and VIP.^19^ PACAP is proven to act as neuroprotective in damaged neurons, it could promote neuronal survival and axonal growth.^20^, ^21^, ^22^, ^23^, ^24^ Instead, it has a controversial role in cancer. In some cases, PACAP has shown to promote tumour growth,^25^, ^26^ whereas it also shown to inhibit tumour growth in other cases.^27^, ^28^ Importantly, functional experiments revealed that PACAP mediated PNI by reprogramming Schwann cells (SCs) in CESC. Collectively, this study deepens our understanding of the PNI mechanism of cervical cancer and may provide novel targets for cervical cancer therapy.

## 2 MATERIALS AND METHODS

### 2.1 Acquisition of histopathology image and molecular data

Diagnostic whole‐slide haematoxylin and eosin stained histopathology images of CESC in the SVS format were downloaded from TCGA database (https://portal.gdc.cancer.gov/). For every image, the corresponding clinical data, patient survival information and raw genomic data were also obtained from TCGA database. Informed consent was obtained by the TCGA consortium. All diagnostic images were publicly available for research purposes, and did not require approval of the institutional review board approval.

### 2.2 Evaluation of perineural invasion

According to previous study,^29^ the degree of PNI was evaluated. In brief, PNI was defined as tumour cells resided within the perineural nerve sheath either in clusters or forming glandular structures. When tumour cells are not resided inside of the nerve sheath but are close to the nerve in the perineural environment, at least one‐third of the circumference of the nerve must be beleaguered by tumour cells to diagnose PNI. Finally, the patients were divided into two groups: PNI (−), without perineural invasion; and PNI(+), perineural invasion.

### 2.3 Differential expression analysis and pathway enrichment analysis

The differential expression analysis of individual genes was analysed using the edgeR Bioconductor package (http://bioconductor.org/packages/edgeR/). For each dataset, samples were divided into two groups, PNI (−) and PNI(+) groups. Raw counts of these samples were extracted and edgeR was used to look for the differentially expressed genes (DEGs) between the two groups. The absolute log2 (fold‐change) ≥1 and *p* ≤ 0.05 were set as restricted condition to identify DEGs. Gene Ontology Enrichment Analysis (GO), Kyoto Encyclopedia of Genes and Genomes (KEGG) and Gene Set Enrichment Analysis (GSEA) software were performed to seek common pathways of CESC in terms of the PNI status as the criterion for sample classification.

### 2.4 Survival analysis and analysis of immune cell infiltration

Disease‐free survival (DFS) and overall survival (OS) were estimated by Kaplan–Meier curves and were compared using the log‐rank test. Hazard ratios (HRs) were calculated using a Cox proportional hazards model. To determine immune cell infiltration in CESC, a total of 28 subpopulations of tumour‐infiltrated leukocytes, including 12 innate immune cells and 16 adaptive immune cells were evaluated as reported previously.^30^ The estimated proportion of individual immune cell types was calculated using single‐sample Gene Set Enrichment Analysis (ssGSEA) in the R package Gene Set Variation Analysis (GSVA).

### 2.5 Patients and tissue samples, immunohistochemistry

We obtained 41 cervical cancer samples from the Department of Pathology, Shanghai Changzheng Hospital, Naval Medical University, after receiving the approval of the Ethical Committee of Shanghai Changzheng Hospital. Informed consent was obtained from each patient. PNI status was assessed directly by a blinded pathologist using haematoxylin and eosin stained histopathology slides. Human tumour specimens were stained with anti‐PACAP rabbit monoclonal antibody (Abcam, ab181205). Horseradish peroxidase‐labelled goat anti‐rabbit secondary antibodies were used (Gene Tech, Shanghai, GK500710). Finally, immunoreactivity was visualized with diaminobenzidine and counterstained by haematoxylin. The staining results were scored by two pathologists blinded to the clinical data.

### 2.6 Cell culture and reagent

Previous studies have shown that HeLa and ME‐180 cells were tend to occur PNI.^17^ Therefore, we selected these two cell lines for further study. Human cervical cancer cell lines HeLa and ME‐180, and Rat Schwann cell line RSC96 were purchased from the Institute of Basic Medical Sciences, Chinese Academy of Medical Sciences (Beijing, China). Cells were cultured in DMEM medium (VivaCell, Shanghai, China), supplemented with 10% fetal bovine serum (FBS) and 1% antibiotics (100 μg/mL streptomycin and 100 units/mL penicillin) at 37°C in a humidified incubator under 5% CO_2_ condition. Recombinant protein was purchased as follows: human recombinant PACAP (MCE, HY‐P0221).

### 2.7 Cell migration assay

Cell migration assay was performed using 8.0 μm pore transparent polyethylene terephthalate inserts (Corning, USA) in 24‐well plates. HeLa, ME‐180 (1 × 10^5^ cells) in 0.2 mL of FBS free media were added to each of the inserts, while RSC96 cells were placed in the bottom with 0.7 mL of medium supplemented with 10% FBS as a chemoattractant. After 24 h for migration assay, the membranes were fixed with 4% polyoxymethylene at room temperature for 20 min and then stained with crystal violet staining solution (Beyotime, Shanghai, China) for 30 min. The cells for each membrane were quantified by counting five random fields at 10× magnification. 3D in‐vitro migration assay was established as described previously.^21^ Briefly, 5 × 10^4^ RSC96 cells were suspended in 20 μL of Matrigel matrix (BD Biosciences, 356234). HeLa‐GFP or ME‐180‐GFP (5 × 10^4^) were suspended in 20 μL of Matrigel, and placed at exact 1 mm distance next to the RSC96‐suspension. In order to exclude the possibility of an unspecifically guided migration of RSC96 cells, a droplet of Matrigel of 20 μL volume containing no cancer cells was placed in opposite directions at the same distance. To enable the formation of the potential signal molecules within the interacting SCs and cancer cells, a 1 mm‐long ECM ‘bridge’ was built in between the suspensions (Figure 3C). And cultures were immobilized by warming to 37°C and cultured in DMEM supplemented with 10% FBS. The co‐cultures were maintained at 37°C under 5% CO_2_ condition for 2 days to observe SCs and cancer cells interactions. Photographic documentation of the ‘migration front’ and the ‘back front’ of SCs suspensions were visualized using laser scanning confocal microscopy (LSM510, Carl Zeiss, Germany).

### 2.8 Repair‐like Schwann cells induction and co‐culture

1 × 10^5^ HeLa or ME‐180 cells were cultured in the lower chamber of 24‐well plates overnight and then 2 × 10^4^ RSC96 cells were seeded on the top chamber (Falcon, USA) and allowed RSC96 to project neurites for 48 h. Neurites were stained with crystal violet staining solution and imaged at 10× magnification. Co‐culture assay was performed as follows: RSC96 cells were seeded onto a 0.4 μm pore Transwell chamber (Corning, USA) to allow cytokines to cross over without cell–cell contact. HeLa or ME‐180 cells were seeded in the bottom of 6‐well plates and co‐cultured with RSC96 cells in 5% CO_2_ at 37°C for 48 h.

### 2.9 Immunofluorescence of human specimen sections

Frozen sections were fixed using 4% paraformaldehyde. All sections were permeabilized and blocked in 3% goat serum, 0.1% Triton X‐100/PBS for 1 h. The following primary antibodies were used for immunofluorescence staining at the indicated dilutions: rabbit anti‐S100B antibody (1:200, CST, 90393S), rabbit anti‐GFAP antibody (1:200, CST, 80788S), rabbit anti‐Vimentin antibody (1:200, CST, 5741S). Sections were incubated overnight at 4°C with primary antibodies diluted in primary antibody dilution buffer. Detection was performed using an appropriate fluorescent secondary antibody (Alexa Fluor 594 [1:500, ABclonal, AS039], Alexa Fluor 488 [1:500, ABclonal, AS053]). Samples were mounted in anti‐fade mounting medium with DAPI. Slides were mounted and examined by confocal microscopy (LSM510; Carl Zeiss, Germany).

### 2.10 RNA sequencing analysis

RNA‐sequencing assay was performed to identify the molecular changes induced by rPACAP proteins. Briefly, total RNA from RSC96 cells with stimulation of PACAP proteins was extracted by Trizol. RNA sequencing analysis was performed by Xuran Biological (Shanghai, China). Gene expression was determined by Fregments Per Kilobase per Million (FPKM) method. Difference in gene expression between groups was analysed by the edgeR software package. DEGs were enriched by GO, KEGG and GSEA.

### 2.11 Western blotting

Western blotting assays were used to verify whether SCs dedifferentiate into unmyelinated phenotype and its corresponding molecular changes. Protein lysates were resolved by electrophoresis on SDS‐PAGE, and proteins were transferred to NC membrane. After blocking in protein‐free rapid blocking buffer (Epizyme, Shanghai, PS108P) for 10 min, the membranes were incubated at 4°C overnight with primary antibodies including PACAP (Abcam, ab181205), GFAP (Abmart, T55424), Vimentin (Abmart, T55134), nestin (Abmart, TD7754), FGF17 (ABclonal, A17864), Cathepsin S (ABclonal, A1874), MMP‐12 (Solarbio, K008189P), E‐Cadherin (CST, 3195S), β‐Tubulin (Abmart, PA4302) and GAPDH (Affinity, AF7021). The antibodies were diluted as recommended by the manufacturers. Bound antibodies were detected using an Odyssey Imaging System (LI‐COR, Biosciences, USA) with DyLight fluorescent dye labelled species‐specific secondary antibodies.

### 2.12 RNA interference and transfection

Three small interfering RNAs (siRNAs) specific for ADCYAP1 as well as scrambled controls were custom‐designed and validated by GenePharma (Shanghai, China). The siRNAs were transfected into HeLa cells and Me‐180 cells using jetPRIME (Polyplus, 101000046) according to the manufacturer's protocol. Cells were transfected with pSLenti‐U6‐shRNA‐CMV‐EGFP‐F2A‐Puro‐WPRE lentiviral particles, constructed by OBiO Technology Corp., Ltd (Shanghai, China), to knockdown the expression of ADCYAP1 in tumour cells.

### 2.13 Statistical analysis

Data were processed using GraphPad Prism 8.0 software and displayed as mean ± standard error of mean (SEM). Comparisons between different groups were using two‐tail unpaired Student's *t‐*test or one‐way analysis of variance (anova). If the *p* < 0.05, the differences were considered significant.

## 3 RESULTS

### 3.1 Integrated characterization of PNI in CESC

**FIGURE 1:**
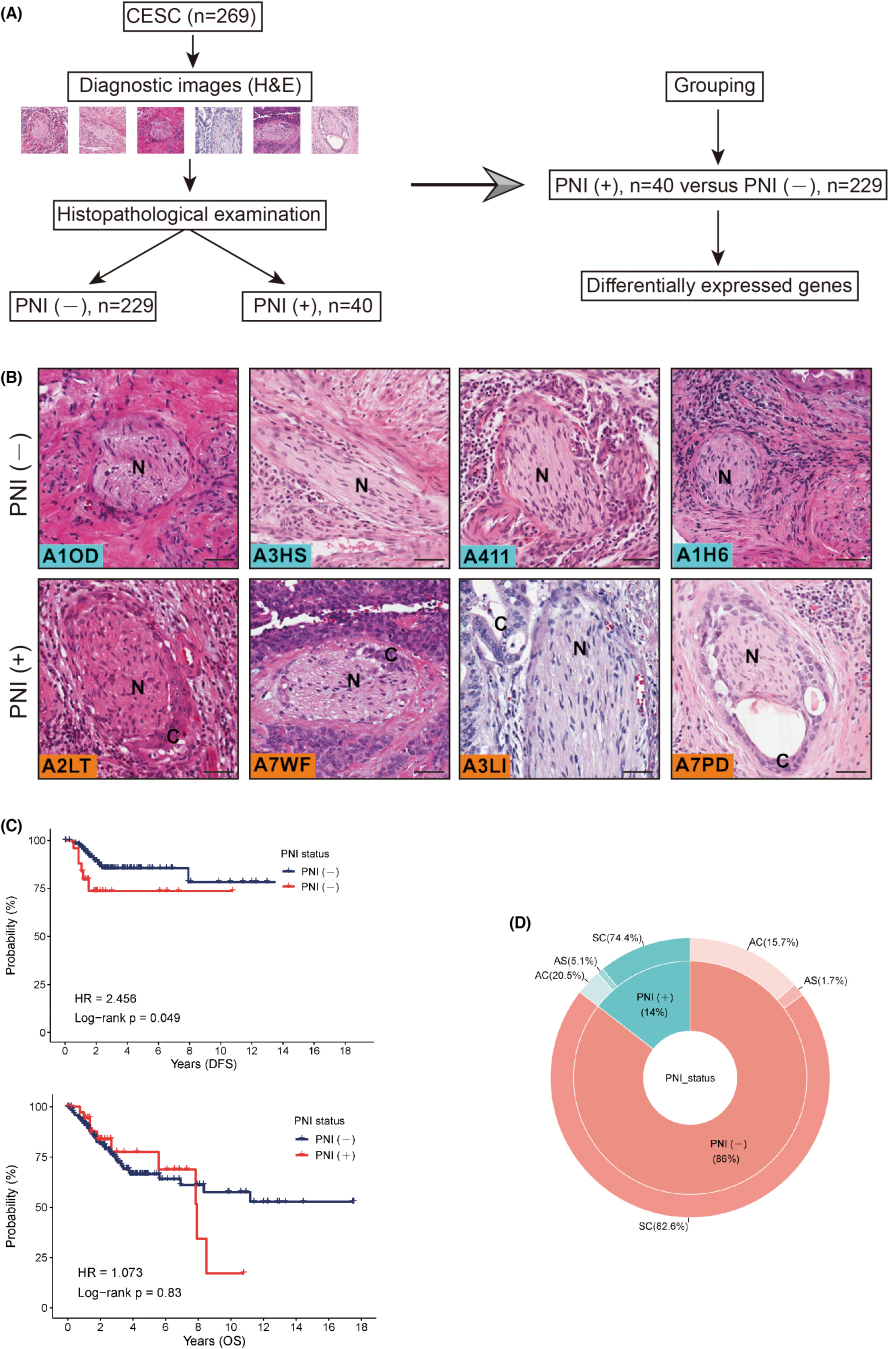
Integrated analysis identifies perineural invasion (PNI)‐associated molecular events in cervical squamous cell carcinoma and endocervical adenocarcinoma (CESC). (A) CESC samples (*n* = 269) in The Cancer Genome Atlas (TCGA) database with diagnostic images were subjected to histopathological examination to define the PNI status. The corresponding transcriptome data of the samples were also analysed. (B) Representative haematoxylin and eosin‐stained images of PNI(−) and PNI(+) groups. Scale bar: 20 μm. (C) Kaplan–Meier disease‐free survival curve and overall survival curve showing the prognostic value of the PNI status in CESC patients from TCGA database (log‐rank test). (D) Composition ratio of pathological types of CESC stratified by PNI status (AC, Adenocarcinomas; SC, Squamous cell neoplasms; AS, Adenosquamous cell carcinomas). The results were not statistically significant.

We obtained haematoxylin and eosin‐stained whole‐slide histopathological images of 269 CESC samples from TCGA database. All images were histopathologically examined by two independent pathologists (Figure 1A). The 269 CESC samples were stratified into two subtypes: PNI(−) (*n* = 229) and PNI(+) (*n* = 40) [see Figure 1A]. Representative haematoxylin and eosin‐stained images of the PNI status are shown in Figure 1B. Kaplan–Meier survival analysis stratified by the PNI status showed that PNI(+) cervical cancer patients may have poorer DFS (HR = 2.456; *p* = 0.049) in TCGA cohort (Figure 1C). However, this difference was not significant for OS. We believed that the small number of PNI(+) samples and the large amount of censored data may have contributed to this bias in the survival analysis. We also found that the PNI status was not associated with the pathological classification of samples (Figure 1D). Table 1 shows the clinicopathological parameters of patients with PNI and matched counterparts. No significant differences were observed between the two subtypes in terms of age, histology, clinical stage and distant metastasis. However, more patients with PNI had lymph metastasis (30.8% vs. 12.8%, *p* < 0.001) and lymphovascular invasion (43.6% vs. 21.3%, *p* = 0.002). This was consistent with the previous notion that PNI(+) cervical cancer patients were more likely to have positive lymph nodes.^12^ Additionally, we evaluated 28 subpopulations of tumour‐infiltrated leukocytes. Interestingly, comparative analysis revealed that patients with the PNI(+) signature had increased infiltration of activated B cells (Figure S1).

**TABLE 1:** Clinicopathological characteristics of patients with perineural invasion (PNI) and their matched group.


PNI status			
	Total (*N* = 269)	PNI(+) (*N* = 39)	PNI(−) (*N* = 230)	*p*‐value
Age (≥45 vs. <45)				
<45	118 (43.9%)	20 (51.3%)	98 (42.6%)	0.404
≥45	151 (56.1%)	19 (48.7%)	132 (57.4%)	
Histology (poor vs. well/moderate)				
Poor (G3)	95 (35.3%)	16 (41.0%)	79 (34.3%)	0.572
Well/moderate (G1‐2)	142 (52.8%)	20 (51.3%)	122 (53.0%)	
Unknown	32 (11.9%)	3 (7.7%)	29 (12.6%)	
Clinical stage (III ~ IV vs. I ~ II)				
I ~ II	201 (74.7%)	33 (84.6%)	168 (73.0%)	0.26
III ~ IV	62 (23.0%)	5 (12.8%)	57 (24.8%)	
Unknown	6 (2.2%)	1 (2.6%)	5 (2.2%)	
Lymph metastasis (present vs. absent)				
Absent (N0)	121 (45.0%)	25 (64.1%)	96 (41.7%)	<0.001
Present (N1)	53 (19.7%)	12 (30.8%)	41 (17.8%)	
Unknown	95 (35.3%)	2 (5.1%)	93 (40.4%)	
Distant metastasis (present vs. absent)				
Absent (M0)	110 (40.9%)	22 (56.4%)	88 (38.3%)	0.103
Present (M1)	10 (3.7%)	1 (2.6%)	9 (3.9%)	
Unknown	149 (55.4%)	16 (41.0%)	133 (57.8%)	
Lymphovascular invasion (present vs. absent)				
Absent	63 (23.4%)	11 (28.2%)	52 (22.6%)	0.002
Present	66 (24.5%)	17 (43.6%)	49 (21.3%)	
Unknown	140 (52.0%)	11 (28.2%)	129 (56.1%)	

### 3.2 Pathway enrichment analysis and differentially expressed genes related to the PNI status

**FIGURE 2:**
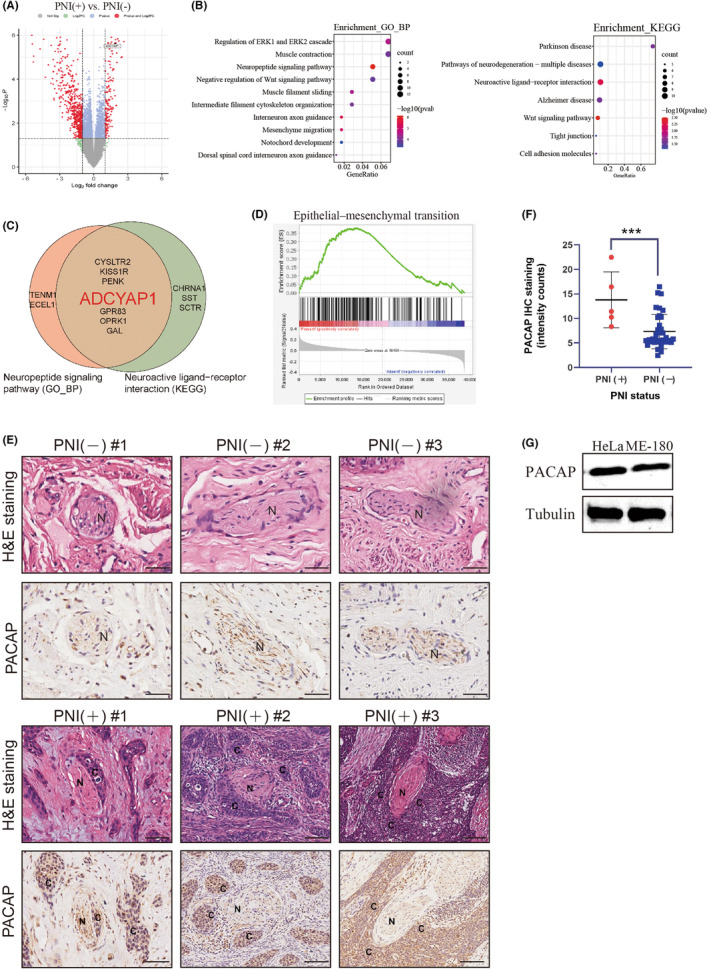
Increased expression of pituitary adenylate cyclase‐activating polypeptide (PACAP) is associated with perineural invasion (PNI) of cervical squamous cell carcinoma and endocervical adenocarcinoma (CESC). (A) Volcano plot of differentially expressed genes (DEGs) related to PNI. ADCYAP1 encoding PACAP was strongly upregulated in the PNI(+) group (log2 fold‐change = 1.952, *p* < 0.0001). (B) GO and KEGG pathway enrichment analysis of DEGs in the PNI(+) group. (C) The same genes were obtained from the intersection of GO and KEGG analyses. ADCYAP1 was selected for further study. (D) Gene Set Enrichment Analysis using hallmark datasets revealed enrichment of epithelial–mesenchymal transition (EMT) in PNI(+) samples. (E) haemotoxylin and eosin staining of cervical cancer tissues showed that tumour cells were morphologically present around the perineurium of the peripheral nerve in PNI(+) samples, whereas tumour cells were not visible in the perineural space in PNI(−) samples. Immunohistochemical staining of PNI(+) cervical cancer tissues revealed diffusely positive staining of PACAP. Conversely, cervical cancer tissues without PNI were generally negative for PACAP (N, nerve; C, cancer cells). Scale bar: 20 μm. (F) Relative intensity of immunohistochemical staining of PACAP in PNI(+) and PNI(−) cervical cancer tissues. *p‐*values were calculated by the Mann–Whitney rank test. (G) Western blotting showing that PACAP was expressed in HeLa and ME‐180 cells. Loading control, Tubulin. ***p < 0.001.

Using mRNA sequencing profiles from TCGA, we identified a spectrum of 85 protein‐coding genes that were significantly upregulated in PNI(+) samples (log2 fold‐change ≥1, *p* < 0.05; Figure 2A). PNI is a complicated process that involves diverse signalling molecules from various signalling pathways. We found that approximately 20% of upregulated DEGs related to the PNI(+) cohort were mainly enriched in neuronal signal‐related pathways (Figure 2B), supporting that our integrated analysis was based on meaningful data in the context of PNI. Neuronal signalling pathways are essential for PNI development. Next, we obtained gene expression profiles related to neuronal signalling pathways in GO and KEGG analyses and then assessed their intersection (Figure 2C). Within the intersection, *ADCYAP1* was drastically upregulated (log2 fold‐change = 1.952, *p* < 0.001, Figure 2A,C).

### 3.3 PACAP and perineural invasion in CESC

Several studies mark the importance of PACAP in SCs‐mediated axonal repair and regeneration^22^, ^24^ Despite extensive studies, the effects of PACAP in PNI remains poorly understood. HeLa and ME‐180 cells were prone to PNI as described previously.^17^ Here, we found that HeLa cells and ME‐180 cells were detected to highly express PACAP (Figure 2G). IHC analyses of human cervical cancer tissues revealed that PACAP immunoreactivity was largely present in tumour tissues that had invaded the nerve (Figure 2E). Meanwhile, correlation analysis suggested that PACAP expression in tumour tissues was closely associated with the PNI status (Figure 2F). Briefly, the high expression of PACAP was positively correlated with cervical cancer PNI.

### 3.4 Cervical cancer cells induce activation of Schwann cells

**FIGURE 3:**
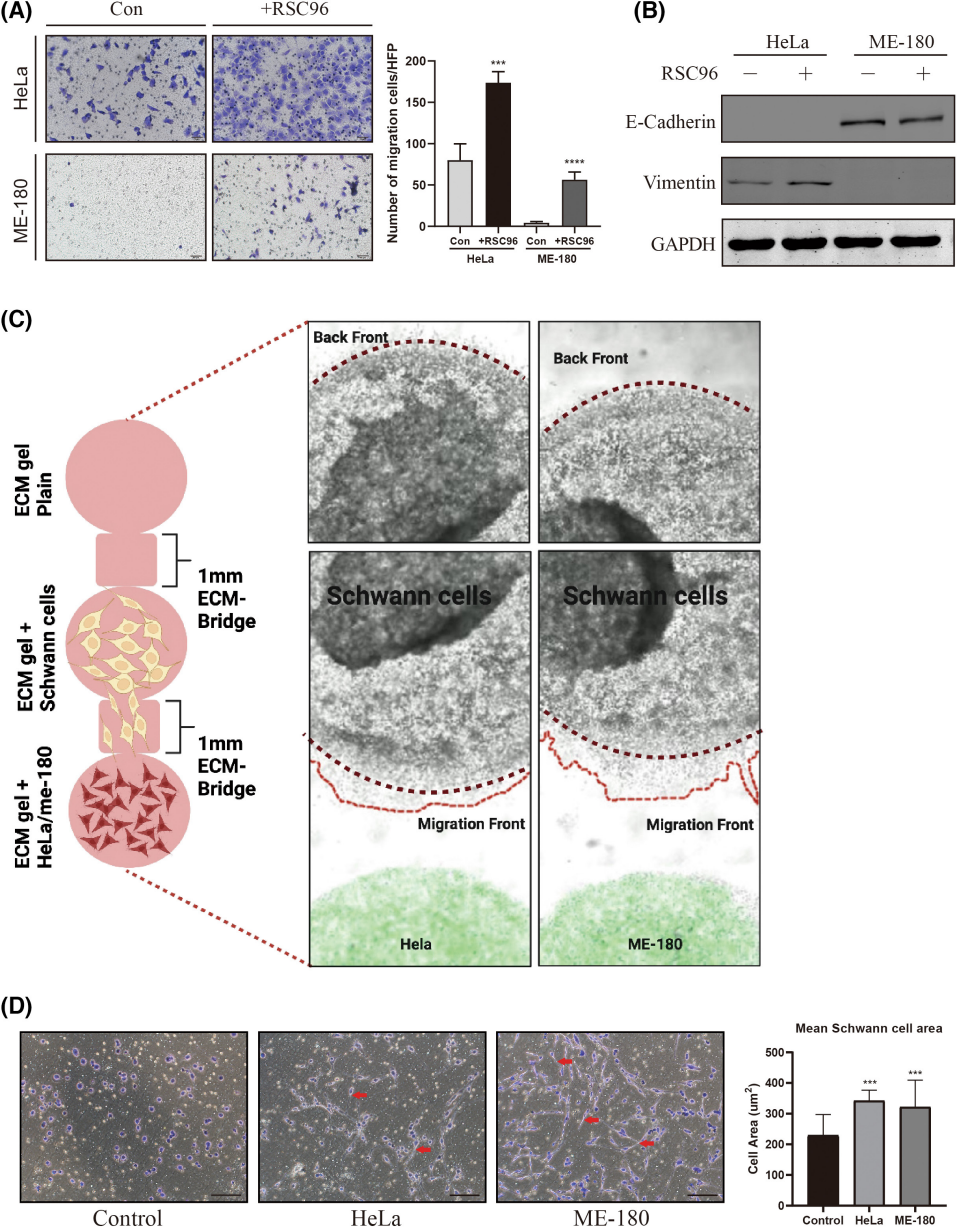
Effect of cervical cancer cells on Schwann cell activation. (A) Schwann cells (SCs) promoted migration of HeLa and ME‐180 cells compared with the control group. Scale bar: 50 μm. (B) EMT markers were altered in HeLa and ME‐180 cells after co‐cultivation with SCs. (C) Schematic view of the 3D in‐vitro migration assay. Specificity of SCs migration towards cervical cancer cells. (D) SCs were co‐cultured with HeLa or ME‐180 cells for 48 h, fixed, and then stained against SCs and the mean cell area was measured. HeLa and ME‐180 cells induced star‐like processes on the Schwann cell surface, and activated SCs were 1.5‐ to 3‐ fold longer than those in the control group. Red arrows indicate the spindle‐shaped processes of hypertrophic SCs. Scale bar: 50 μm. ***p < 0.001, ****p < 0.0001.

Astrogliosis is a dominant event in the central nervous system (CNS) following injury.^31^ Reactive astrocytes are characterized by proliferation and cellular hypertrophy, and acquire a star‐like conformation.^31^, ^32^ SCs are a type of glial cell in the peripheral nervous system and respond similarly to astrocytes during nerve trauma. However, the morphological changes of SCs in nerve–cancer interactions are largely unexplored. Indeed, co‐culture SCs with HeLa or ME‐180 cells resulted in elongation of the cellular morphology of SCs, which is highly reminiscent of repair‐like SCs in the injury response (Figure 3D). Correspondingly, the cellular area of SCs co‐cultivated with cervical cancer lines HeLa (342.2 ± 12.10 μm^2^) and ME‐180 (322.0 ± 24.18 μm^2^) was significantly greater than that of control SCs (229.5 ± 22.63 μm^2^, Figure 3D). These findings indicated that cervical cancer cells exerted glial cell‐activating effects. Additionally, we used a 3D migration‐assay in which RSC96 cells were simultaneously confronted with HeLa cells or ME‐180 cells on one side and with control suspension on the other side (Figure 3C). After 2 days of co‐culture, before cervical cancer cells even started with their migration towards glial cells, SCs had already exhibited a very early and highly targeted migration to cervical cancer cells. Based on these observations, SCs could obviously activated and chemoattracted by cancer cells, and hijacked by them to trigger PNI. In this study, we also verified that SCs markedly enhanced HeLa and ME‐180 cell migration in a contact‐independent co‐culture system (Figure 3A). Western blotting confirmed that SCs induced EMT of HeLa and ME‐180 cells as evidenced by downregulation of E‐Cadherin in ME‐180 cells and upregulation of Vimentin in HeLa cells (Figure 3B).

### 3.5 Cervical cancer cells induce GFAP and Vimentin expression in Schwann cells

**FIGURE 4:**
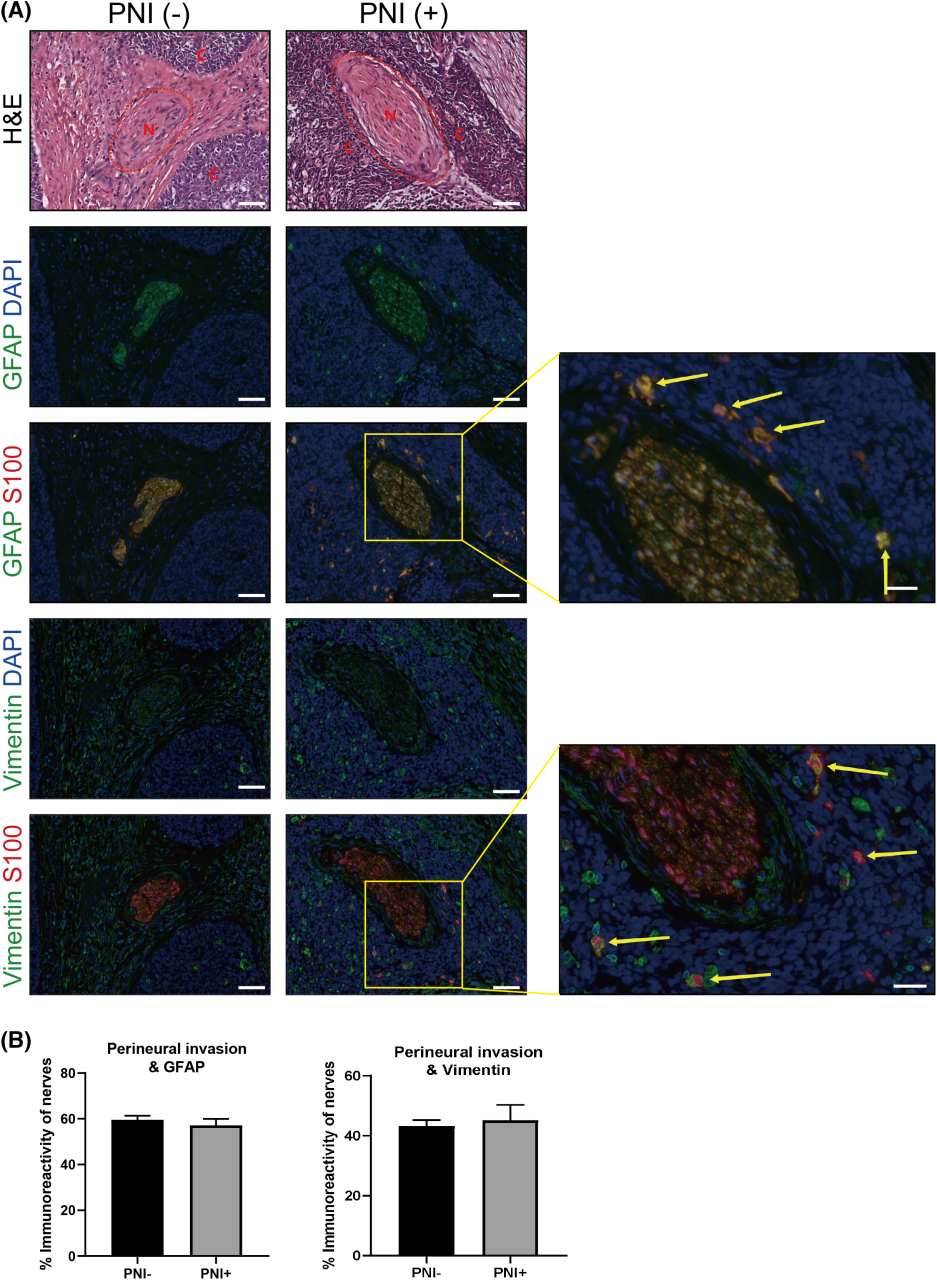
Dedifferentiated Schwann cells at neoplastic cell sites before the onset of cervical cancer invasion. (A) Representative nerve sections from cervical cancer patients stained for GFAP (green, second row), Vimentin (green, fourth row) and S100B (red) and corresponding haematoxylin and eosin‐stained sections. A different tissue section from the same patient in an area without perineural invasion (PNI) was used as the control. Scale bar: 50 μm. Higher magnification images showed that GFAP+ or Vimentin+ SCs had infiltrated the area of cancer cells. Scale bar: 20 μm. Yellow arrows denote examples of activated SCs in the tumour stroma. (B) No statistical significance between the presence of PNI and immunofluorescence reactivity of GFAP and Vimentin.

**FIGURE 5:**
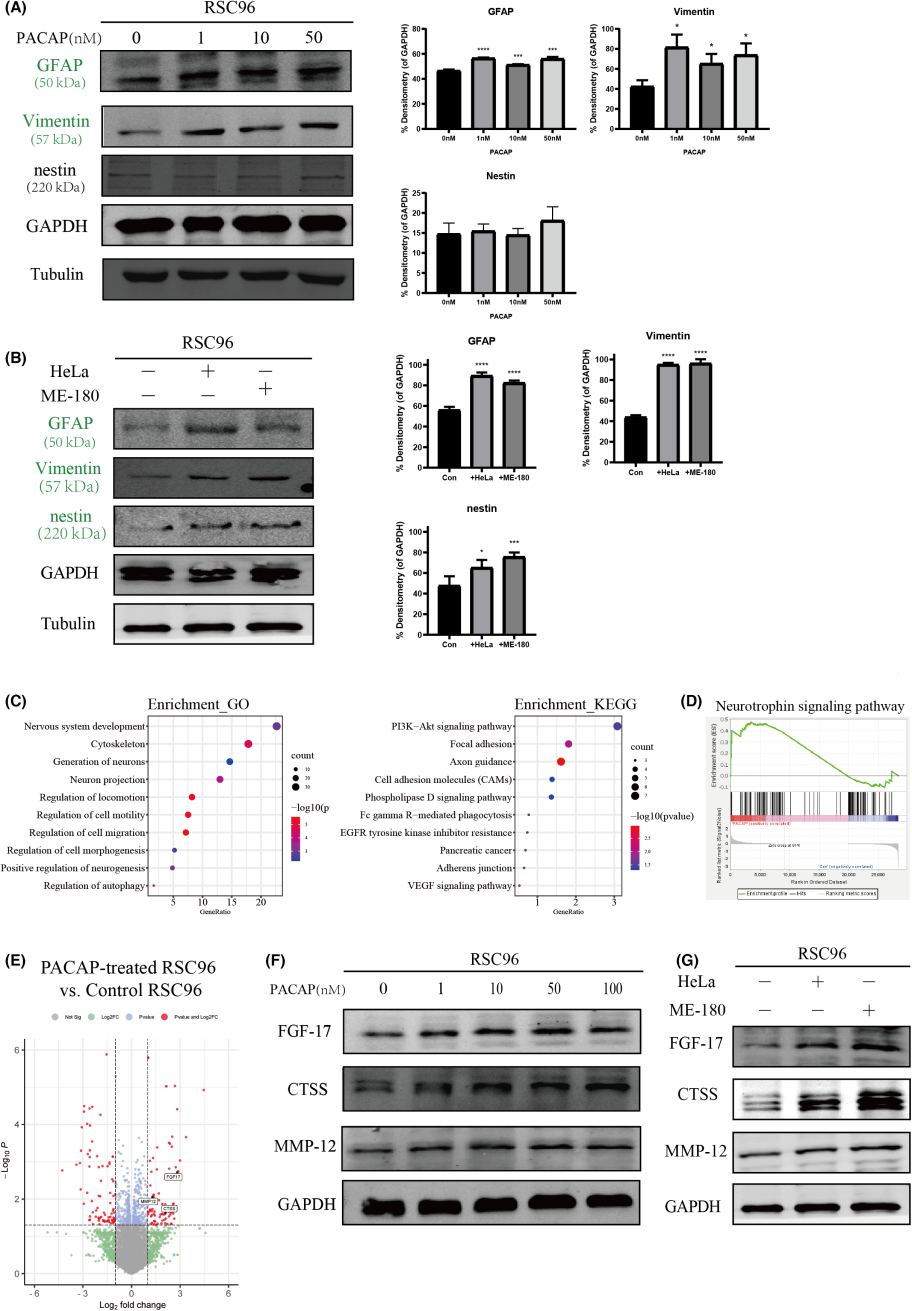
Crosstalk between Schwann cells and cervical cancer cells. (A) Schwann cells (SCs) were exposed to various concentrations of rPACAP followed by protein isolation to assess activation markers by western blotting. (B) Cervical cancer cells significantly promoted GFAP, Vimentin and nestin expression of SCs in the co‐culture system. (C) GO and KEGG enrichment analyses of differentially expressed genes (DEGs) induced by rPACAP in SCs. (D) Gene Set Enrichment Analysis showed a correlation between PACAP and the neurotrophin signalling pathway in SCs treated with rPACAP. (E) Volcano plot of DEGs in SCs after rPACAP treatment. (F) SCs were treated with various concentrations of rPACAP for 48 h and then FGF17, CTSS and MMP‐12 expression was measured by western blotting. (G) HeLa and ME‐180 cells upregulated FGF17, CTSS and MMP‐12 expression in SCs. * *p* < 0.05, *** *p* < 0.001, **** *p* < 0.0001.

Second, we examined the potential glial cell‐activating properties of cervical cancer cells. Addition of human recombinant PACAP to the growth medium of SCs increased the intracellular levels of glial fibrillary acidic protein (GFAP) and Vimentin (intermediate filaments), but did not affect nestin (neuronal marker) expression (Figure 5A). However, the upregulation of GFAP and Vimentin was independent of direct contact of cervical cancer cells with nerves, because the presence of PNI was not associated with any major change in immunoreactivity for GFAP [PNI(−): 60.57% ± 2.1%; PNI(+): 58.52% ± 1.87%; Figure 4A,B] or Vimentin [PNI(−): 48.41% ± 7.3%; PNI(+): 49.66% ± 7.4%; Figure 4A,B]. Notably, in human cervical cancer specimens with PNI, we found close associations between GFAP+ or Vimentin+ SCs and cervical cancer cells. GFAP+ or Vimentin+ SCs were closely associated with cancer cells by infiltrating in tumour stroma (Figure 4A, higher magnification images). These observations were consistent with previous studies indicating that SCs migrate towards cancer cells before the onset of cancer migration towards peripheral neurons.^33^ Such a discovery has further overturned the dogma that cancer cells first attack nerves. In the co‐culture system of cervical cancer cells and SCs, signals from the cancer cells markedly enhanced the expression of GFAP, Vimentin and nestin in SCs (Figure 5B). Thus, our results indicated that cervical cancer cells had a strong glial cell‐activating property that induced GFAP and Vimentin expression in SCs partially via the PACAP paracrine signalling.

### 3.6 FGF17, Cathepsin S and MMP‐12 are responsible for promotion of PNI by repair‐like Schwann cells

To reveal the mechanism by which PACAP promotes PNI, we performed genome‐wide transcriptomic analysis of RSC96 cells after treatment with human recombinant PACAP (100 nM) for 48 h. GO and KEGG analyses as well as GSEA revealed that PACAP had a significant implication in signalling pathways of the cytoskeleton, neuron projection, cell migration and axonal guidance (Figure 5C,E). These data further indicate that PACAP plays a major role in PNI. Previous studies have revealed that crosstalk among reactive astrocytes and neurons during gliosis involve paracrine and autocrine influences via cytokines and growth factors, especially IL‐1, NGF and FGFs.^31^ Analysis of upregulated DEGs induced by rPACAP showed that fibroblast growth factor 17 (FGF17) expression was significantly upregulated in the treatment group (log2 fold‐change = 2.89, *p* = 0.0019, Figure 5E). The FGF/FGFR signalling network plays a critical role in the development of invasive cervical cancer.^34^ Therefore, FGF17 might play a critical role in PNI of cervical cancer. Apart from chemoattractants secreted by SCs induce PNI, it is also plausible that SCs facilitate cancer cell dissemination along nerves by an additional mechanism. PNI requires an adaptable microenvironment to degrade the extracellular matrix (ECM).^16^ Our analysis of SCs treated with rPACAP revealed increased expression of Cathepsin S (CTSS) and MMP‐12 (log2 fold‐change = 2.04 and 1.35, *p* = 0.025 and 0.042, respectively; Figure 5E). Western blotting showed that rPACAP induced expression of FGF17, CTSS and MMP‐12 in SCs (Figure 5F). Moreover, SCs displayed increased protein expression of FGF17, CTSS and MMP‐12 after co‐cultivation with cervical cancer cells (Figure 5G).

## 4 DISCUSSION

Multiple types of cancer are actively promoted by the nerve microenvironment. Recent studies have demonstrated that infiltrating nerves in tumour microenvironment and denervation of the tumour may alter its growth, suggesting involvement of the nervous system in tumorigenesis and tumour progression. Furthermore, for several types of cancer, PNI may indicate aggressive behaviour and a poor prognosis. However, little attention has been focused on the mechanisms of PNI in cervical cancer. Understanding the cellular and molecular mechanisms involved in nerve participation in PNI of cervical cancer and identifying the cells involved are crucial to choose suitable operation and to develop new therapeutic approaches. Using multiomics and clinical data of CESC from TCGA, this study characterized PNI‐associated molecular changes, including mRNA gene expression, which might be pioneering. Integrative analysis and functional experiments highlighted a unique role of PACAP secreted from cancer cells in PNI of CESC by activation of SCs.

SCs are glial cells in the peripheral nervous system, which wrap around axon where they exhibit remarkable plasticity and the ability to modulate pathology. SCs respond rapidly and develop into a radical phenotypic change after nerve injury. This process has two major components: the reversal of myelin differentiation and the appearance of new phenotypes.^35^ Molecules that characterize dedifferentiation of SCs include p75NTR and GFAP,^35^ similar to activated astrocytes in CNS during injury.^31^, ^32^, ^36^ Meanwhile, demyelination would trigger extensive and striking SCs elongation and branching to form long, parallel processes.^37^ Recent studies reported that SCs could be used by tumour cells to form a repair phenotype similar with that in the process of nerve regeneration, thereby augmenting cancer metastasis.^18^, ^38^, ^39^ During this process, myelinated SCs transdifferentiated into so‐called repair Schwann cells (rSCs) and re‐express GFAP, Vimentin and many other molecules.^40^ Although there are studies showed that tumour cells use SCs dedifferentiation properties for their own favour.^38^ Until recently, apart from nerve injury and regeneration, the roles of SCs were underestimated in cancer neuroscience. This study indicates that SCs are participants rather than bystanders in PNI of cervical cancer. Tumour‐activated SCs dedifferentiate into a non‐myelinated repair‐like subtype of SCs to trigger PNI.

Various cancer types may express divergent SCs activator molecules such as IL‐6,^32^ IL‐1β,^18^ and CXCL12.^33^ Our experiments showed that PACAP may has a strong glial activation capacity. PACAP is an important factor to promote peripheral neural regeneration and regulate the inflammatory response following injury. After peripheral nerve injury, expression of PACAP upregulates in SCs and it is released at the site of nerve injury to promote regeneration of the nerve stump and regulate inflammation.^21^, ^41^ PACAP also exerts protective effects on neurons by regulating glial cells activation,^23^ during this process, PACAP increases GFAP expression in reactive astrocytes.^42^ Beyond that, many studies have suggested that PACAP expression links to cancer. For example, the PACAP expression is known to be overexpressed in breast cancer,^26^ lung cancer,^43^ and prostate cancer.^44^ On the contrary, it has also shown that PACAP suppresses tumour growth in glioblastoma,^45^ multiple myeloma,^46^ and cervical cancer.^27^ Taken together, these facts are consistent with a previous study that PACAP has both pro‐tumour and anti‐tumour effects on tumour cell.^25^ Therefore, its role in cancer neuroscience may also be multifaceted.

We found that activated SCs after co‐cultured with cervical cancer cells displayed the same cardinal features of reactive astrocytes in the central nervous system, that is, upregulation of intermediate filament proteins (GFAP and Vimentin), cellular hypertrophy (star‐like cellular conformation change) and increased secretion of cytokines and proteinases. Moreover, the expression of GFAP and Vimentin was also upregulated when SCs were treated with rPACAP. Hence, this glial activation appeared to depend on PACAP paracrine signalling in vitro. While our findings are encouraging, it was difficult to determine the exact relationship between tumour‐derived PACAP and activated SCs. Due to the short coding sequence of ADCYAP1, we could not specifically knock out PACAP in cervical cancer cells. Nevertheless, our findings have provided a solid foundation for further functional verification and clues to unravel the reversal of SCs phenotypes in PNI of cervical cancer. Overall, we showed that tumour‐activated SCs exhibit the same cardinal characteristics as rSCs after nerve injury, and acquisition of this phenotype may partially depend on PACAP paracrine signalling.

Mechanistically, PACAP signalling that induces PNI entails crosstalk between cervical cancer cells and SCs. Our data showed that PACAP induced FGF17 expression. Fibroblast growth factor signalling has a tumour‐promoting role. Apart from effects on proliferation and survival, FGFs are also involved in the regulation of cell migration and angiogenesis in various cancer types.^47^ Just like PACAP could stimulate SCs to produce proteases to clear cellular debris after peripheral nerve injury,^48^ SCs also promote rapid metastasis of tumour cells via secreting abundant proteases capable of degrading matrix molecules and cell adhesions. After ECM degradation, green channels form, which are conducive to rapid metastasis of tumour cells. We demonstrated that PACAP (either paracrine or recombinant) induced CTSS and MMP‐12 expression in SCs. CTSS promotes cancer cell invasion through numerous mechanisms including ECM degradation, cleavage of cellular adhesion molecules and stimulation of angiogenesis.^49^ Additionally, proteins degraded by cathepsin are important components of the perineurium, which facilitate tumour cell metastasis along nerves through cathepsin‐mediated destruction of the protective perineurium.^50^ Among the factors involved in PNI, matrix metalloproteinases (MMPs), especially MMP‐2 and MMP‐9, are believed to be essential for collagen degradation and drive cancer cells to disseminate along nerves.^16^, ^17^ In this study, we found upregulated expression of MMP‐12 in activated SCs. Thus, PACAP facilitates PNI via two mechanisms: (1) PACAP upregulates expression of FGF17 that serves as a chemoattractant along intracervical neural bundles and (2) PACAP facilitates ECM breakdown by upregulating CTSS and MMP‐12 expression along an axon.

Our study also has some limitations. First, it is still unclear whether PACAP has a dominant paracrine effect in PNI of CESC. Second, we cannot specifically interfere with the expression of PACAP in cervical cancer cells due to the coding sequence of ADCYAP1 is too short. Finally, murine sciatic nerve model of PNI could not be performed due to the current inability to target the corresponding receptor on glial cells in vivo.

**FIGURE 6:**
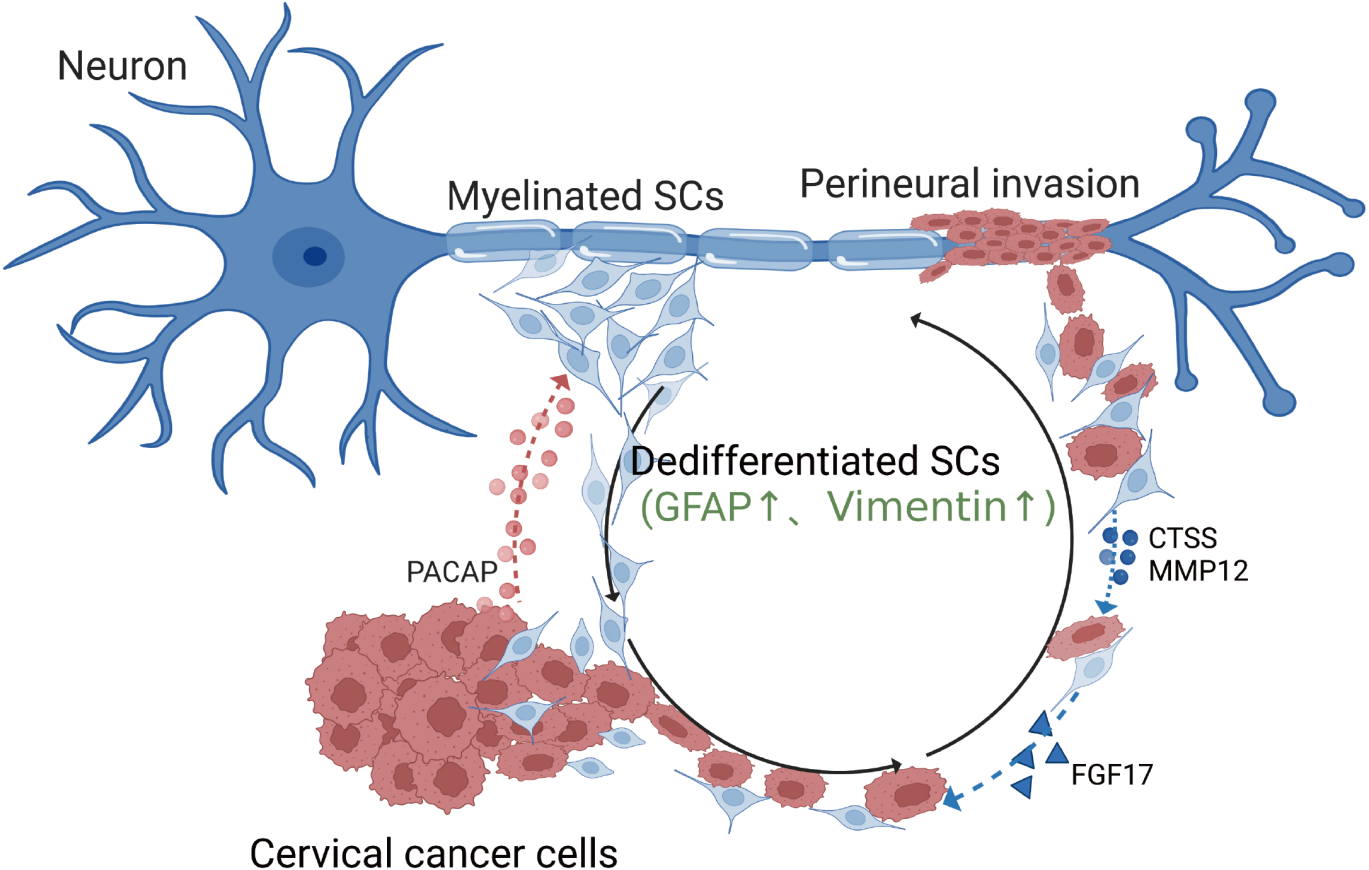
A model of neural–tumour crosstalk mediated by the neuropeptide pituitary adenylate cyclase‐activating polypeptide (PACAP) in cervical cancer. Schwann cells (SCs) dedifferentiate and migrate towards the tumour stroma under the influence of PACAP secreted from cervical cancer cells. These dedifferentiated SCs exhibit star‐like cellular conformation change and produce FGF17, CTSS and MMP‐12 to promote tumour metastasis. These steps ultimately lead to perineural invasion.

In summary, the present study demonstrated that tumour‐derived PACAP activates SCs in the microenvironment of cervical cancer. Activated SCs exhibit the same cardinal characteristics as rSCs during peripheral nerve injury, and this phenotype is partly mediated by PACAP paracrine signalling. In turn, SCs activation leads to PNI of cervical cancer by secreting FGF17, CTSS and MMP‐12 (Figure 6). These factors serve as chemoattractants of tumour cells or degrade the ECM, leading to an alteration of the cancer‐neural niche. Therefore, developing specific inhibitors to interrupt the cancer–Schwann cell axis may be a novel and promising therapeutic approach for PNI of cervical cancer.

## AUTHOR CONTRIBUTIONS

**Guoqiang Chen:** Data curation (lead); formal analysis (lead); investigation (lead); methodology (lead). **Zhen Zheng:** Data curation (equal); formal analysis (equal); investigation (equal); methodology (equal). **Hao Sun:** Data curation (equal); formal analysis (equal); methodology (equal). **Jiahao You:** Investigation (supporting); methodology (supporting). **Jing Chu:** Data curation (supporting); formal analysis (supporting). **Jinghai Gao:** Investigation (supporting); methodology (supporting); project administration (supporting). **Lei Qiu:** Conceptualization (equal); investigation (lead); project administration (equal). **Xiaojun Liu:** Funding acquisition (lead); project administration (lead).

## FUNDING INFORMATION

The research was supported by grants from National Social Science Foundation of China (2023‐SKJJ‐B‐038 to Xiaojun Liu), Special Research Program on Aging and Maternal and Child Health of Shanghai Municipal Health Commission (2020YJZX0210 to Xiaojun Liu).

## CONFLICT OF INTEREST STATEMENT

The authors have no conflict of interest.

## Supporting information


Data S1.

## Data Availability

Data available on request from the authors.
