# Supplementary material for: Dedifferentiated Schwann cells promote perineural invasion mediated by the PACAP paracrine signalling in cervical cancer
Source: J Cell Mol Med. 2023 Oct 13;27(23):3692–705. doi: 10.1111/jcmm.17897 (PMC10718160; doi:10.1111/jcmm.17897)
Supplement: Supplementary file 1 — Data S1. [file JCMM-27-3692-s001.docx]

**Supplementary Materials:**

**
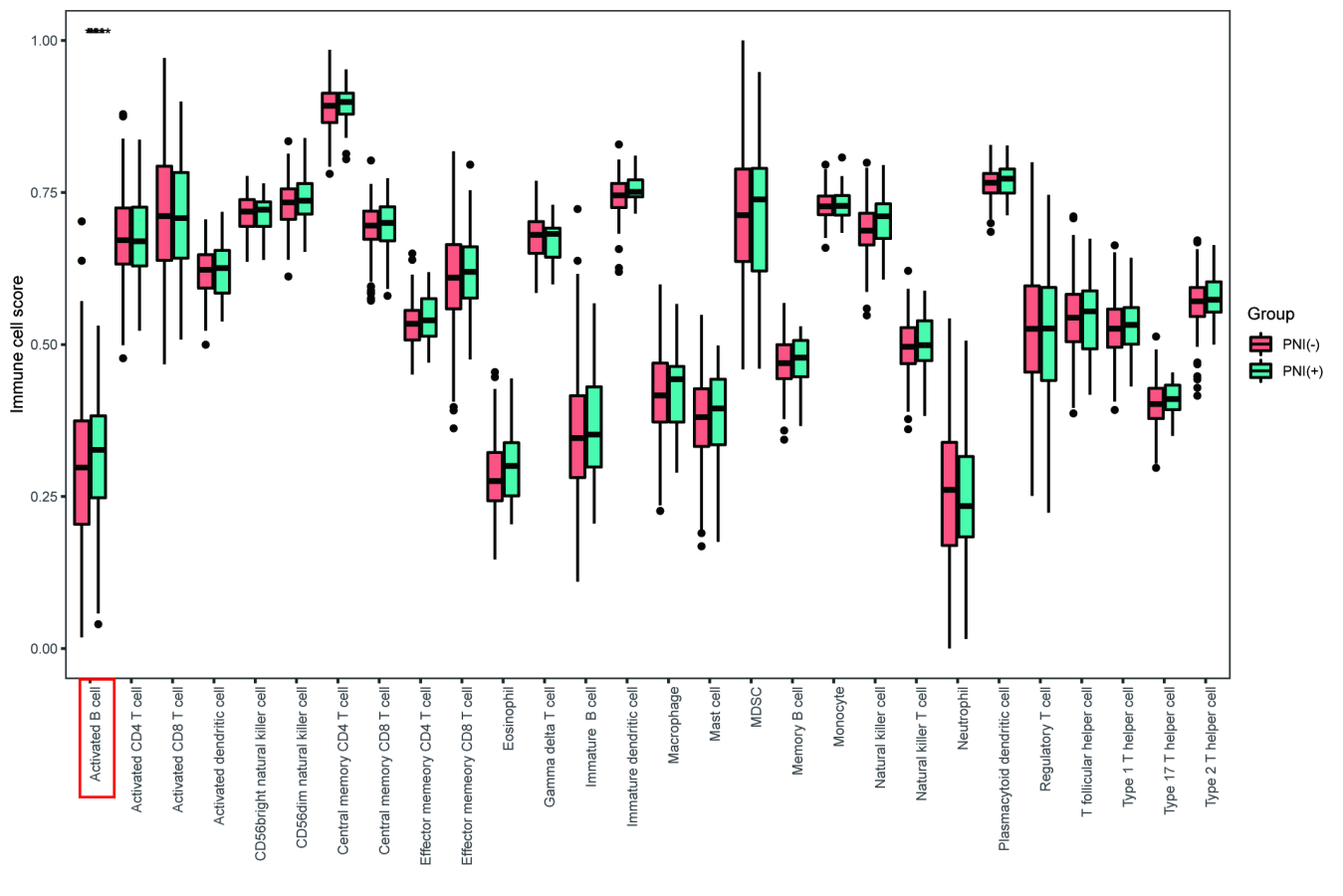
**

**Figure S1.**Comparison of 28 immune cell subtypes between the PNI (+) and PNI (-) groups. Activated B cell was marked by red frame. **** *P* <0.0001.
